# Dynamic assessment of community resilience in China: empirical surveys from three provinces

**DOI:** 10.3389/fpubh.2024.1378723

**Published:** 2024-04-19

**Authors:** Cunling Yan, Xiaoyu Liu, Ning Zhang, Ying Liu, Bingjie Wang, Caihong Sun, Yunli Tang, Yue Qi, Bingyan Yu, Luhao Zhang, Ning Ning

**Affiliations:** ^1^Department of Social Medicine, School of Health Management, Harbin Medical University, Harbin, China; ^2^Department of Complaint Management, Harbin Medical University Cancer Hospital, Harbin, China; ^3^Think Tank of Public Health Security and Health Reform of Heilongjiang Province, Harbin, China

**Keywords:** community resilience, China, dynamic assessment, CART, empirical survey

## Abstract

**Background:**

Strengthening the construction of community resilience and reducing disaster impacts are on the agenda of the Chinese government. The COVID-19 pandemic could alter the existing community resilience. This study aims to explore the dynamic change trends of community resilience in China and analyze the primary influencing factors of community resilience in the context of COVID-19, as well as construct Community Resilience Governance System Framework in China.

**Methods:**

A community advancing resilience toolkit (CART) was used to conduct surveys in Guangdong, Sichuan, and Heilongjiang provinces in China in 2015 and 2022, with community resilience data and information on disaster risk awareness and disaster risk reduction behaviors of residents collected. The qualitative (in-depth interview) data from staffs of government agencies and communities (*n* = 15) were pooled to explore Community Resilience Governance System Framework in China. Descriptive statistics analysis and *t*-tests were used to investigate the dynamic development of community resilience in China. Hierarchical regression analysis was performed to explore the main influencing factors of residential community resilience with such socio-demographic characteristics as gender and age being controlled.

**Results:**

The results indicate that community resilience in China has improved significantly, presenting differences with statistical significance (*p* < 0.05). In 2015, connection and caring achieved the highest score, while disaster management achieved the highest score in 2022, with resources and transformative potential ranking the lowest in their scores in both years. Generally, residents presented a high awareness of disaster risks. However, only a small proportion of residents that were surveyed had participated in any “community-organized epidemic prevention and control voluntary services” (34.9%). Analysis shows that core influencing factors of community resilience include: High sensitivity towards major epidemic-related information, particular attention to various kinds of epidemic prevention and control warning messages, participation in epidemic prevention and control voluntary services, and formulation of epidemic response plans. In this study, we have constructed Community Resilience Governance System Framework in China, which included community resilience risk awareness, community resilience governance bodies, community resilience mechanisms and systems.

**Conclusion:**

During the pandemic, community resilience in China underwent significant changes. However, community capital was, is, and will be a weak link to community resilience. It is suggested that multi-stages assessments of dynamic change trends of community resilience should be further performed to analyze acting points and core influencing factors of community resilience establishment at different stages.

## Introduction

In recent years, uncertain public disasters and disturbances, including sudden public health emergencies (such as infectious disease) and natural disasters (such as earthquakes, floods, and hurricanes) (1, 2), have been witnessed worldwide, presenting a trend of normalization. Human casualties and economic losses caused by various disasters should not be underestimated. Disaster risks have become the biggest obstacle to sustainable development (3). The outbreak of COVID-19 in 2020 is a major global public health emergency with far-reaching and profound impacts on the physical and mental health of human beings, global economic development, and international exchanges (4). Even today, its influences can still be experienced with varying degrees (5, 6). Although production and daily life have returned to their normal tracks around the world, human beings are still facing threats from uncertain disasters. The establishment of such resilience capabilities as alerting, resistance, adaptation, and recovery in disaster scenarios has become an issue that needs to be urgently addressed in the world (6, 7).

Community plays a crucial role as the foundational unit of society, with the level of integration within the community directly influencing the collaborative capacity of its members and the effectiveness of collective efforts in disaster management. The concept that strong social integration is indispensable for societal development (8), which also holds significant implications for community level of disaster management. As a basic defense unit in disaster management (9), the community is not only a direct subject confronting disasters but also an important place for carrying out disaster prevention and responses, with its actions affecting the overall level of disaster prevention and mitigation (10). During the COVID-19 pandemic, the Chinese government implemented a series of measures at the community level to curb the spread of the virus (11). These measures include community lockdowns with different degrees, strict restrictions on entering and leaving communities and resident outdoor activities (12), and grid-based and carpet-style management (13). Community residents actively participated in epidemic prevention and control and transformed individual involvement into collective efficacy to address disaster events faced by communities collectively (14). The practice has shown that strong community resilience constitutes a foundation supporting good operational performances of communities during the epidemic. Additionally, the construction of community resilience serves as a strategic approach towards disaster management.

In 1973, the theory of resilience was first applied to the field of ecology and the concept of resilience has been enriched by researchers in the disciplines (15). With the degradation of the ecological environment and the increasing frequency of disasters, community disaster resilience has gradually become one of the mainstream research subjects of disaster resilience (2, 16). The concept of community resilience is complex, with diverse interpretations across disciplines. Nevertheless, as its core, it can be conceptualized that community resilience reflects the abilities of communities to respond and adapt to the impacts of various external disturbances and disasters, including self-organizing abilities, adjustment abilities under pressure, and learning and adaptation abilities of communities (17, 18). Community resilience is a set of dynamic positive attributes rather than a simple static result. Community is a complex social system, and the assessment of community resilience is regarded as a key factor in reducing disaster risks (19). Cutter et al. (20) constructed the index of community resilience baseline characteristics and established The Baseline Resilience Indicators for Communities (BRIC) model, which has been widely utilized to assess community resilience through a series of secondary indicators (17, 20, 21). Based on the prioritizing risk management and reducing system vulnerability, Orencio et al. (22) developed the Local Disaster Resilience Index (LDRI), which used Delphi technology to explore the vulnerability standards and related factors affecting coastal communities through the AHP method (22). Pfefferbaum and other scholars jointly constructed and modified The Communities Advancing Resilience Toolkit (CART) based on their practical experiences (23), which can be used to acquire basic information about communities, identify advantages and challenges of community resilience establishment, and reassess the capacity building of community resilience after a disaster (23–25). The development of community resilience theory and evaluation models has significantly improved the efficacy of community disaster management strategies.

The assessment of community resilience research within the framework of comprehensive disaster prevention and mitigation has become a foundational paradigm, utilizing both qualitative and quantitative methodologies for description and analysis (26). At the operational level, CART is one of the most influential questionnaires, which is a tool for assessing community disaster resilience capacity based on theory and evidence, and it has already been verified with good reliability and validity by various studies at home and abroad (27–29). After the COVID-19 outbreak, researchers have mostly utilized CART to measure community resilience. Siska et al. (3) identified that Transformative Potential and Information and Communication domains differed significantly between Indonesian and Malaysian communities, with Indonesian demonstrated a higher score on Transformative Potential, while Malaysian indicated a higher score on Information and Communication. Empirical research, such as the study conducted by Josephine et al. (29) in Rural Australia, revealed that the primary community resilience strengths were related to the Connection and Caring, whereas the challenges related to Resources. Furthermore, the findings identified that community resilience affected the implementation of a mental health promotion program, and lack of communication and leadership were key barriers to the implementation of the program. In another study with collected 650 valid questionnaires from three citys in China, Zhang (2) indicated that domains such as Information and Communication, and Connection and Caring scored higher. Additionally, the findings identified that participating in volunteer responder groups and exerting the community disaster risk reduction activities were the biggest impact on community resilience. Current studies on the status quo of community resilience are mostly based on cross-sectional surveys without employing a longitudinal comparative analysis.

China is a populous country with vast territory, and various uncertain disaster events occur in this country frequently. Strengthening the establishment of community resilience and reducing disaster impacts are on the agenda of the Chinese government. In 2020, the Chinese government officially introduced the concept of “resilient city” (30), and in 2021, it explicitly stated the goal to build “resilient cities” (31). In China, the evaluation of community resilience primarily relies on qualitative method in the development of index dimensions, including literature reviews, case studies, and policy analyses. Consequently, the majority of pertinent research remains confined to the theoretical level, while existing quantitative studies still limited to cross-sectional surveys. In 2015, our research team conducted an assessment on community resilience in three representative provinces in China. However, with the significant impacts of the COVID-19 pandemic on grassroots communities, previous community resilience in China could have changed. Therefore, the dynamic development trend of community resilience in China deserved to in-depth study.

Thus, based on the Chinese national conditions and real disaster situations, this study aims to describe and analyze the dynamic evolution process of community resilience in China, and explore the key influencing factors in the context of COVID-19 through the surveys of community resilience in three provinces in China before the outbreak of COVID-19 and at the late stage of the pandemic, then construct Community Resilience Governance System Framework in China.

## Materials and methods

### Data sources

In order to ensure that the study samples can reflect the characteristics of regional geographic location and economic development level, this study selected community residents in three regions in China, namely, Guangdong Province (east region), Sichuan Province (west region), and Heilongjiang Province (central region) based on Chinese Yearbook statistics data. Empirical surveys were conducted twice on community residents in these three regions during the periods from April to June in 2015 and from November to December in 2022 to collect information on community resilience levels during different stages.

A random sampling method was employed by researchers during the first round of empirical surveys conducted in 2015. Based on the conditions of urban population and area distribution in these three provinces, one major city, one medium-sized city, and one town city in each province were randomly selected. Then, two communities were randomly selected from each selected city. With the guidance of community managing staff, uniformly-trained investigators carried out face-to-face interviews with community residents and conducted on-site questionnaire surveys under the informed consent of the respondents.

The COVID-19 pandemic has greatly impacted grassroots community resources and community networks. In response to disasters, communities must make adjustments, adaptations, and changes (2). This could result in changes in community resilience. Against the backdrop of the COVID-19 pandemic, a second round of empirical surveys was conducted in this study at the end of the year 2022. Community residents in Guangdong, Sichuan, and Heilongjiang were still selected as respondents of these surveys. Due to the national pandemic prevention and control policies implemented at that time, the daily traveling of community residents was affected to varying degrees. Considering the limitation during this specific period, we applied a non-random sampling method and conducted questionnaire surveys with the support of the Wenjuanxing platform. Wenjuanxing^1^ is an online questionnaire distribution and collection platform that has been widely used in various surveys and studies. During the data collection process, online-trained local community administrators invited local residents to fill out questionnaires by scanning QR codes through WeChat with their mobile phones. Respondents would browse through questionnaire instructions and requirements before entering the questionnaire item pages. They were required to click and confirm an informed consent key before they could enter the official questionnaire-filling interface. It is sure that respondents could quit the survey at any time when filling out the questionnaires.

In addition, in order to address the limitations of the non-random sampling in 2022 and ensure the validity and reliability in assessing the multifaceted nature of resilience, an in-depth interview was conducted onsite. Based on the similarity external environment of the unified governance structure of the Chinese government, the impact of the pandemic on the communities and the top-down and uniform community-based administrative measures, as well as the unified social network and cultural context, the differences among communities could be mitigated. Therefore, 15 interviewees were purposively selected based on their roles and experience in government agencies and local communities in Heilongjiang province. Three senior researchers with extensive experience in qualitative research conducted all interviews in-person and one-on-one to ensure the feedback to be independent and confidential. Meanwhile, the researchers developed a semistructured interview protocol to ensure that all relevant topics were covered. Topics covered were: (1) elements of community preparedness and response to disasters; (2) the network structure of community resilience and (3) how to enhance community resilience.

### Data quality control

In order to select representative research samples, no limitation on respondents’ ages was imposed during the questionnaire survey process. However, it was ensured that all objects surveyed were independent adults capable of critical thinking (≥18 years old). It was ensured that all investigators engaged in those two rounds of surveys had undergone uniform training and examination and were familiar with the survey methods, target populations, and measurement contents. Meanwhile, they were required to possess good language communication and expression skills. Questionnaires with obvious logic errors, uncompleted and completion time less than 2.5 min, as well as multiple questionnaires submitted from a single IP address, were excluded (It takes at least 3 min to complete the whole questionnaire at a normal rate of reading and answering questionnaire items). A total of 2,506 valid questionnaires were collected during the first round of surveys, and a total of 1,936 valid questionnaires were collected during the second round of surveys.

## Measures

### Community resilience

CART is a tool developed by Pfefferbaum and other scholars based on extensive research and continuous practical modifications (32). In early versions of the toolkit, four domains correlated with community resilience, namely, connection and caring, resources, transformative potential, and disaster management, were identified. Some studies revealed that significant promoting effects of “information and communication” on those four domains of CART, as well as their importance in community resilience and disaster management, can be identified in the use of the scale (23). Therefore, information and communication were also incorporated into the assessment model of community resilience, thus forming a five-factor model for assessing community resilience (25). Our research team was the first to introduce CART into China after translating it under the normal process, and it has been proven that CART presents good reliability and validity among Chinese sample populations (27).

Based on different social, cultural and economic backgrounds and community development status in China, a Chinese version of CART was used in this study to evaluate community resilience in China, especially the level of community resilience against the backdrop of the COVID-19 pandemic. Five domains and twenty-four items were included in the scale. Among them, five items were contained in the domain of connection and caring, five items were contained in the domain of resources, six terms were contained in the domain of transformative potential, four items were contained in the domain of disaster management, and four items were contained in the domain of information and communication. A Likert five-point scale was used to measure the degree of agreement. A score of each domain was obtained with the sum of the scores of its items, and a total CART score was obtained with the sum of the scores of all domains. With the consideration of Chinese cultural attributes, in this study, responses of “strongly agree”, “agree”, and “neither disagree nor agree” in each item were regarded as positive responses, while responses of “strongly disagree” and “disagree” in each item were regarded as negative responses.

### Disaster risk awareness

Risk awareness refers to the intuitive judgment of the public on risks (33). Knowledge plays an important role in risk awareness. The public utilized knowledge to access the impacts of the novel coronavirus and took corresponding behavioral actions. In disaster literature, it shows that risk awareness can significantly affect the willingness of the public to prepare for emergencies (34). During the COVID-19 pandemic, risk awareness of communities was an important component in reducing disaster risks.

In this study, three question items were designed to evaluate the risk awareness of community residents on the COVID-19 pandemic. These question items included: “I think it is highly possible that the pandemic will outbreak in surrounding areas,” “I am very sensitive to the reports of major epidemic-related information,” and “I will always pay special attention to warning messages about various epidemic prevention and control.” Answer options to all these questions were set as 1 and 0, with 1 representing a positive response (agree) and 0 representing a negative response (disagree). All variables were set as dummy variables.

### Disaster risk reduction behaviors

Reducing disaster risks is a key part of resilience building (35). At the initial stage of the pandemic outbreak, unknown diseases and various uncertainties caused tremendous panic among the public, which is still fresh in their memories. Communities also fell into chaos due to a lack of supplies, personnel, and funding, as well as no timely information and communication (36). During the late stage, with the carrying out of such various disaster prevention and mitigation actions as increased public awareness of the disease, rational regulation and control of the government, sufficient supply of community supplies, and voluntary participation of residents, community resilience was effectively restored and developed (12).

In this study, three question items related to disaster risk reduction behaviors during the pandemic were designed to test the disaster risk reduction behaviors of people. These question items include: “Have you stored any relevant emergency supplies for the pandemic (such as masks, disinfectants, and medicines)?” “Have you ever participated in any community-organized voluntary services for epidemic prevention and control?,” and “Have you and your family formulated any epidemic response plans?.” The corresponding disaster risk reduction behaviors of participants were: (1) preparing emergency supplies at home for the pandemic, (2) participating in voluntary services organized by communities for epidemic prevention and control, and (3) formulating epidemic response plans. Answer options for all these three questions were set as 1 and 0, with 1 representing a positive response (yes) and 0 representing a negative response (no). All variables were set as dummy variables.

### Socio-demographic characteristics

Common socio-demographic characteristics of those two rounds of empirical surveys include gender, age, marital status, education status, and years lived in their neighborhood. Among them, ordinal categorical variables were set as follows: (1) Age: ≤30 = 1, 30–50 = 2, >50 = 3; (2) Education status: primary school and below = 1, middle/high school = 2, College and above = 3; (3) Years lived in their neighborhood: <1 year = 1, 1–5 years = 2, ≥5 years = 3. Nominal categorical variables were set as follows: (1) Gender: male = 1, female = 0; (2) Marital status: married = 1, other = 0. All variables were set as dummy variables.

### Data analysis

IBM SPSS Statistical version 26.0 was used for data processing and analysis. Descriptive statistics was used to analyze the socio-demographic characteristics, community resilience, disaster risk awareness, and disaster risk reduction behaviors. A statistical analysis using *t*-tests was performed on the dynamic development status of community resilience (*t*-tests are applicable to data following a normal distribution). A hierarchical regression model was constructed with community resilience as a predictive variable, socio-demographic characteristics as control variables, and disaster risk awareness and disaster prevention and mitigation behaviors as core explanatory variables to investigate the main influencing factors of community resilience. A significance level of 0.05 was used for all statistical tests.

The qualitative interview data were categorised and analysed thematically by three researchers independently using triangulation method. The coding framework was developed inductively from the data. The initial coding used open coding (codes derived directly from the data) and theoretical coding, The initial codes were then refined to produce a smaller set of themes and a consensus was reached among researchers.

## Results

### Socio-demographic characteristics of the participants

A total of 4,442 valid questionnaires were collected during those two rounds of empirical surveys. A total of 2,506 questionnaires were collected for the surveys conducted in 2015, with an average age of 41.2 ± 13.5 years among all respondents of these surveys. Male, married, holding a college degree or above, and with years lived in their neighborhood exceeding five years accounted for 47.0%, 83.8%, 53.7%, and 64.4% of all participants of these surveys, respectively. A total of 1,936 questionnaires were collected for the surveys conducted in 2022, with an average age of 35.9 ± 9.8 years among all participants of these surveys. Male, married, holding a college degree or above, and with years lived in their neighborhood exceeding five years accounted for 42.7%, 55.1%, 82.5%, and 55.3% of all participants of these surveys, respectively (see Table 1).

**Table 1:** Descriptive analysis of socio-demographic characteristics (*N* = 4,442).

Characteristics	In 2015 (*N* = 2,506)		In 2022 (*N* = 1,936)	
	Frequency	Percentage (%)	Frequency	Percentage (%)
Gender				
Male	1,177	47.0	826	42.7
Female	1,329	53.0	1,110	57.3
Age (years)				
≤30	584	23.3	1,067	55.1
30 ~ 50	1,664	66.4	755	39.0
>50	258	10.3	114	5.9
Marital status				
Married	2099	83.8	1,067	55.1
Unmarried or others	407	16.2	869	44.9
Education status				
Primary school and below	270	10.8	26	1.3
Middle or high school	889	35.5	313	16.2
College and above	1,347	53.7	1,597	82.5
Years lived in their neighborhood (year)				
<1	256	10.3	30	1.5
1 ~ 5	631	25.3	836	43.2
≥5	1,603	64.4	1,070	55.3

## Community resilience

According to the scoring status of five domains in the CART scale, it can be seen that the average scores of community resilience in these two rounds of surveys are (2.95 ± 0.706, 3.64 ± 0.698) respectively. The highest-scoring domains in these two rounds of surveys are slightly different from each other, with “connection and caring” (3.18 ± 0.748) being the highest-scoring domain in 2015 and “disaster management” (3.79 ± 0.746) being the highest-scoring domain in 2022. There is no change in the lowest-scoring domain during those two rounds of surveys, with “resources” being the lowest-scoring domain, followed by “transformative potential.”

According to the scoring status of twenty-four items in the CART scale, it can be seen that item 4, “People in my community help each other,” achieved the highest scores during those two rounds of surveys, with agreement proportions of 90.4% and 93.6% obtained in 2015 and 2022, respectively. The lowest-scoring items during those two rounds of surveys are slightly different from each other. Item 7, “My community has the resources it needs to take care of community problems,” scored the lowest in 2015, with an agreement proportion of 62.0%. In contrast, item 8, “My community has effective leaders,” scored the lowest in 2022, with an agreement proportion of 87.9%.

Data collected in this study follow a normal distribution and present homogeneity of variance. Therefore, *t*-tests can be used to compare data in two groups. From Table 2, it can be clearly seen that community resilience achieved significantly higher scores in 2022 than those in 2015, and *t*-test analysis indicated that differences between these two groups are statistically significant (*p* < 0.05) (see Table 2).

**Table 2:** Descriptive and comparative analysis of core community resilience items by domains of CART.

Domains and Items	In 2015		In 2022		*t* value
	Mean (SD)	NPR (%)	Mean (SD)	NPR (%)	
Connection and Caring	3.18 (0.748)		3.61 (0.755)		−18.757*
People in my community feel like they belong to the community	3.08 (0.920)	78.3	3.45 (0.972)	89.0	−13.219*
People in my community are committed to the well-being of the community	3.00 (0.928)	74.1	3.48 (0.949)	89.6	−16.708*
People in my community have hope about the future	3.15 (0.938)	79.9	3.62 (0.964)	91.7	−16.475*
People in my community help each other	3.44 (0.879)	90.4	3.84 (0.893)	93.6	−14.872*
My community treats people fairly no matter what their background is	3.26 (0.932)	84.0	3.67 (0.964)	92.3	−14.236*
Resources	2.82 (0.863)		3.51 (0.808)		−27.414*
My community supports programs for children and families	2.82 (1.148)	63.4	3.49 (1.036)	87.6	−19.877*
My community has the resources it needs to take care of community problems	2.71 (0.967)	62.0	3.43 (1.038)	88.6	−23.935*
My community has effective leaders	2.79 (0.996)	65.9	3.38 (1.019)	87.9	−19.544*
People in my community can get the services they need	2.87 (0.988)	70.1	3.62 (0.946)	92.0	−25.433*
People in my community know where to go to get things done	2.91 (1.021)	71.1	3.66 (0.954)	92.0	−25.096*
Transformative Potential	2.83 (0.823)		3.58 (0.788)		−30.559*
My community works with organizations and agencies outside the community to get things done	2.75 (0.972)	64.9	3.47 (0.963)	88.7	−24.437*
People in my community communicate with leaders who can help improve the community	2.85 (0.981)	68.1	3.60 (1.005)	91.6	−24.965*
People in my community work together to improve the community	2.91 (0.988)	70.3	3.69 (0.971)	92.7	−26.105*
My community looks at its successes and failures so it can learn from the past	2.83 (0.939)	68.5	3.54 (0.982)	90.9	−24.304*
My community develops skills and finds resources to solve its problems and reach its goals	2.82 (0.945)	68.1	3.57 (1.028)	90.6	−25.322*
My community has priorities and sets goals for the future	2.81 (0.961)	68.1	3.60 (0.977)	91.6	−26.735*
Disaster Management	2.97 (0.929)		3.79 (0.746)		−31.829*
My community tries to prevent disasters	2.95 (1.049)	70.7	3.82 (0.911)	91.9	−29.011*
My community actively prepares for future disasters	2.94 (1.044)	70.0	3.82 (0.913)	93.2	−29.481*
My community can provide emergency services during a disaster	2.98 (1.018)	72.4	3.78 (0.930)	92.0	−26.746*
My community has services and programs to help people after a disaster	2.99 (1.014)	73.1	3.74 (0.968)	90.4	−24.849*
Information and Communication	3.00 (0.900)		3.76 (0.773)		−29.639*
My community keeps people informed about issues that are relevant to them	2.97 (1.093)	70.8	3.80 (0.968)	89.6	−26.450*
If a disaster occurs, my community provides information about what to do	3.00 (0.995)	73.2	3.81 (0.949)	91.0	−27.512*
I get information/communication through my community to help with my home and work life	2.98 (1.006)	72.4	3.69 (0.999)	88.6	−23.067*
People in my community trust public officials	3.06 (1.018)	76.0	3.75 (0.923)	91.5	−23.481*
Overall Community Resilience	2.95 (0.706)		3.64 (0.698)		−32.169*

SD, standard deviation; NPR (%), percentage of positive responses (including neither disagree nor agree); *p < 0.05.

### Disaster risk awareness and disaster risk reduction behaviors of the participants

Risk awareness is an important component in reducing disaster risks, and reducing disaster risks is a key part of resilience building. Therefore, in the construction of community resilience, risk awareness and disaster risk reduction behaviors of residents should be considered. Relevant items were added in the surveys conducted at the end of 2022 to investigate disaster risk awareness and disaster risk reduction behaviors of residents. From Table 3, it can be seen that residents generally presented a high level of disaster risk awareness, with a proportion of up to 92.5% for residents who “pay special attention to various warning information of epidemic prevention and control.” However, in terms of disaster risk reduction behaviors, a small proportion of residents “had ever participated in any community-organized voluntary services for epidemic prevention and control,” accounting for only 34.9% of all correspondents of the surveys.

**Table 3:** Descriptive analysis of disaster risk awareness and disaster risk reduction behaviors.

Variables	Frequency	Percentage (%)
Disaster Risk Awareness		
I think it is highly possible that the pandemic will outbreak in surrounding areas		
Yes	1,515	78.3
No	421	21.7
I am very sensitive to the reports of major epidemic-related information		
Yes	1744	90.1
No	192	9.9
I will always pay special attention to warning messages about various epidemic prevention and control		
Yes	1790	92.5
No	146	7.5
Disaster Risk Reduction Behaviors		
Have you stored any relevant emergency supplies for the pandemic (such as masks, disinfectants, and medicines)		
Yes	1,590	82.1
No	346	17.9
Have you ever participated in any community-organized voluntary services for epidemic prevention and control		
Yes	676	34.9
No	1,260	65.1
Have you and your family formulated any epidemic response plans		
Yes	1728	89.3
No	208	10.7

### Analysis of the influencing factors of community resilience

In the hierarchical regression model with overall community resilience as a predictive variable, disaster risk awareness and disaster prevention and mitigation behaviors as core explanatory variables, considering the influence of confounding factors, socio-demographic characteristics as control variables, the fitness of the models are appropriate. The control layer model presented an *R*^2^ value of 0.076, with *F* value of 31.779 (*p* < 0.001). With the addition of core explanatory variables, the *R*^2^ value increased to 0.213, with *F* value of 47.224 (*p* < 0.001). In addition, all of the VIF values are all less than 5. The results indicate that 13.7% of the variation in community resilience can be explained by variables of disaster risk awareness and disaster prevention and mitigation behavior. In terms of influencing directions and significance of explanatory variables, the model constructed with scores of all domains as predictive variables presented consistent output results and comprehensive scores.

After controlling for socio-demographic factors, the following results can be obtained from Table 4: (1) the stronger disaster risk awareness of residents results in a higher score of community resilience. Residents who “are very sensitive to major epidemic-related information” and “pay special attention to various warning messages of epidemic prevention and control” will achieve relatively high scores in their community resilience. (2) Effective disaster risk reduction behaviors can raise the level of community resilience. Residents who “have participated in voluntary services of epidemic prevention and control” and “formulated epidemic response plans” will achieve relatively high scores in their community resilience.

**Table 4:** Hierarchical regression results for community resilience.

Variables	Overall		Community Resilience Domains									
			Connection and Caring		Resources		Transformative potential		Disaster management		Information and Communication	
	Control layer	Core variable layer	Control layer	Core variable layer	Control layer	Core variable layer	Control layer	Core variable layer	Control layer	Core variable layer	Control layer	Core variable layer
Gender	−0.014 (−0.443)	−0.010 (−0.337)	−0.006 (−0.035)	0.013 (0.082)	−0.146 (−0.805)	−0.098 (−0.573)	−0.315 (−1.483)	−0.269 (−1.344)	0.214 (1.592)	0.201 (1.565)	−0.079 (−0.564)	−0.080 (−0.608)
Age	−0.172** (−5.545)	−0.148** (−5.170)	−0.933** (−5.574)	−0.813** (−5.213)	−0.964** (−5.348)	−0.846** (−4.998)	−0.931** (−4.412)	−0.793** (−3.986)	−0.622** (−4.647)	−0.536** (−4.202)	−0.669** (−4.809)	−0.570** (−4.353)
Marital status	−0.372** (−10.251)	−0.251** (−7.329)	−1.890** (−9.632)	−1.267** (−6.798)	−2.140** (−10.132)	−1.498** (−7.407)	−2.313** (−9.349)	−1.575** (−6.626)	−1.215** (−7.741)	−0.803** (−5.269)	−1.368** (−8.391)	−0.885** (−5.648)
Education status	0.150** (4.048)	0.113** (3.250)	0.860** (4.287)	0.667** (3.524)	0.553* (2.562)	0.377 (1.835)	0.909** (3.594)	0.736** (3.050)	0.776** (4.838)	0.606** (3.916)	0.505** (3.029)	0.328* (2.061)
Years lived in their neighborhood	0.135** (4.551)	0.112** (4.055)	0.896** (5.561)	0.775** (5.169)	0.682** (3.934)	0.554** (3.406)	0.844** (4.156)	0.693** (3.624)	0.401** (3.111)	0.325** (2.651)	0.430** (3.214)	0.336** (2.663)
Highly possible that the pandemic will outbreak in surrounding areas	/	0.055 (1.565)	/	0.372* (1.962)	/	0.448* (2.176)	/	0.322 (1.332)	/	0.138 (0.887)	/	0.030 (0.186)
Sensitive to the reports of major epidemic-related information	/	−0.257** (−4.852)	/	−1.441** (−4.991)	/	−1.099** (−3.508)	/	−1.274** (−3.461)	/	−1.151** (−4.876)	/	−1.214** (−5.007)
Always pay special attention to warning messages about various epidemic prevention and control	/	−0.356** (−5.918)	/	−1.919** (−5.861)	/	−1.574** (−4.429)	/	−1.955** (−4.682)	/	−1.510** (−5.639)	/	−1.592** (−5.788)
Have you stored any relevant emergency supplies	/	−0.049 (−1.326)	/	−0.197 (−0.970)	/	−0.212 (−0.961)	/	−0.024 (−0.092)	/	−0.427* (−2.572)	/	−0.327 (−1.922)
Have you ever participated in any community-organized voluntary services	/	−0.184** (−6.120)	/	−0.943** (−5.776)	/	−1.167** (−6.589)	/	−1.207** (−5.797)	/	−0.442** (−3.311)	/	−0.649** (−4.734)
Have you and your family formulated any epidemic response plans	/	−0.555** (−11.761)	/	−2.825** (−10.997)	/	−3.166** (−11.361)	/	−3.655** (−11.160)	/	−1.665** (−7.926)	/	−2.017** (−9.347)
*R*^2^	0.076	0.213	0.076	0.204	0.066	0.182	0.065	0.175	0.055	0.148	0.050	0.163
△*R*^2^	0.076	0.137	0.076	0.128	0.066	0.116	0.065	0.111	0.055	0.093	0.050	0.113
*F* value	31.779***	47.224***	31.875***	44.762***	27.362***	38.910***	26.703***	37.152***	22.276***	30.333***	20.085***	33.972***

*p < 0.05, **p < 0.01, and ***p < 0.001.

### The results of in-depth interview

**Figure 1:**
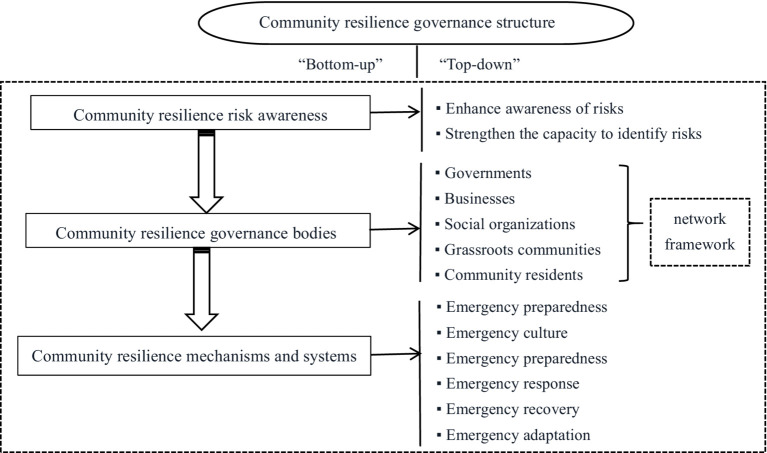
Community resilience governance system framework in China.

Of the 15 individuals who participated in the in-depth interview, 6 came from government agencies and 9 came from community institution. All of the interviewees had working experience of epidemic prevention and control, and all of them had engaged in administrative work for more than 5 years. Based on the theory of The Community Disaster Risk Management and the concept of Risk Governance, and combined with the political context and actual practices of community epidemic prevention and control in China, we try to construct Community Resilience Governance System Framework in China, which includes three elements: community resilience risk awareness, community resilience governance bodies, community resilience mechanisms and systems (see Figure 1).

## Discussions

### The overall level of community resilience in China has been significantly improved

The survey results in this study show that after the outbreak of the COVID-19 pandemic, community resilience in China has undergone significant changes. The score of each item of community resilience during the late stage of the pandemic, as well as its overall score, is all above 3 (that is, neither agreement nor disagreement) but is lower than 4 (that is, agreement). These scores are significantly higher than those achieved during the first round of surveys in 2015. In addition, “connection and caring” is the top-scoring domain obtained from the surveys in 2015, while it is replaced by “disaster management” as the top-scoring domain in 2022. After the outbreak of the pandemic, the Chinese government quickly and decisively implemented potent prevention and control measures, adhering to the notion of “people first and life first.” Meanwhile, the entire nation united together closely. Working towards one direction, Chinese people carried out effective disaster management and epidemic responses. Grassroots communities actively employed measures against the epidemic and prepared for the potential risks of the epidemic. These measures included mutual assistance among community residents, significant investment in community epidemic prevention resources, timely disclosure of epidemic information, and providing door-to-door services for residents under quarantine and control. Therefore, people’s lives were fully protected and respected. These measures represent a good manifestation of disaster management in the case of an epidemic, constituting a good cornerstone of community resilience building and fully reflecting the trust of people in the government. The intensity of community resilience can be measured with a percentage of consistency.

Strength for community resilience are identified as survey items with the highest percentages of consistency. Meanwhile, weakness for community resilience are identified as survey items with the lowest percentages of consistency (15, 37). In this study, item 4, “People in my community help each other, “was identified as a strength item for community resilience. Neighborhood support is a key component of social capital, and its role in enhancing community resilience has been widely recognized and promoted (25, 38, 39). Item 7, “My community has the resources it needs to take care of community problems,” and item 8, “My community has effective leaders,” were identified as weakness item for community resilience during those two rounds of surveys. This indicates that resources of communities were, are, and will be weak links that require special attention in the building of community resilience.

### The establishment and development of social capital in community resilience should be fully strengthened

Although community resilience building in China has achieved some progress, we must be clear that the weak point of community resilience building in China in the past and present is the severe lack of community capital. From the perspective of community resilience, social capital refers to the willingness and abilities of community members to actively engage in and contribute to activities promoting the common goals of communities (40). Communities with abundant social capital normally present greater resilience (41, 42). Powerful and responsible community leaders are among the most important factors in addressing public health crises (43), playing a crucial role in promoting close communication among community residents and establishing good partnerships with non-governmental organizations (44). Some studies show that community human resources under normalized epidemic prevention and control have the problems of insufficient reserves, irrational allocation, and weak integration capacities (45). Furthermore, community leaders lack sufficient handling and leading skills for emergency events. Empirical survey results in this study also indicate that the item “My community has effective leaders” ranked the lowest in scoring. Therefore, strengthening the construction of community talent and fostering excellent community leaders are key points for the development of social capital in community resilience in the present and future China.

Besides the human capital of communities, social cohesion and community resources are also key points in the building of community resilience. The study results show that the items “My community has the resources it needs to take care of community problems,” “My community works with organizations and agencies outside the community to get things done,” and “People in my community feel like they belong to the community” all ranked relatively low in their scoring. Under the influence of the traditional Confucian cultural thought of “a neighbor in close proximity is more advantageous than a distant relative,” mutual assistance and support among neighbors during the COVID-19 pandemic were fully reflected. The impact of a disaster can undermine interpersonal relationships and diminish the sense of communal cohesion (46). Some problems, such as an inadequate sense of identity and belonging among community residents, persisted. In addition, investment in community resources was insufficient, and cooperation degrees between communities and external organizations were relatively low.

### Disaster risk awareness, disaster risk reduction behaviors are key factors that need to be focused on in community resilience

The study results show that compared with static socio-demographic characteristics (such as gender, marital status, education status, and years lived in their neighborhood), high levels of disaster risk awareness of residents and their active participation in disaster risk reduction have a more significant impact on community resilience. Therefore, this study has focused on these two influencing factors: disaster risk awareness and disaster risk reduction behaviors.

The vigilance of residents towards risks is one important aspect of community resilience enhancement. Existing studies have shown that individuals with higher perception of risks present higher levels of resilience (47). COVID-19 is a new infectious disease that exerts uncertain potential impacts among residents. Community residents with higher risk awareness are more interested in relevant information on major epidemic outbreaks and actively pay attention to various warning information on epidemic prevention and control. When facing epidemic disasters, these community residents will positively perceive and evaluate community resilience. Bennett et al. (48) established a model linking risk awareness with risk mitigation behaviors. This model assumes that community residents with higher risk awareness are more likely to take preventive measures based on official information to reduce the probability of risk occurrence, presenting high evaluation levels of community resilience.

Participation in disaster risk reduction behaviors at the community level is a key influencing factor in community resilience enhancement. This study shows that residents engaging in community voluntary services are more likely to develop a positive view of the capacities of their communities against disasters, which is consistent with the research results obtained by Pfefferbaum et al. (25). Residents participating in community voluntary activities present enhanced community collective awareness, as well as improved abilities against crisis events (49, 50). Involvement in voluntary services against the COVID-19 pandemic has not only strengthened the internal cohesion within the voluntary union but also promoted interactions and communication among community residents and improved communities’ emergency response capabilities. It is the core target of community resilience to develop targeted plans to address current risks and potential public emergencies in the future (51). The key point of the preventive strategy lies in the involvement of the community and its residents in the whole process. Community residents who actively engage in epidemic risk assessment and epidemic response plan formulation will be presented with more opportunities to proactively obtain information, knowledge, and resources related to the pandemic (52). This finding could indicate that positive community interaction is a potential factor in individual perception of community resilience.

### Exploring community resilience governance system framework in China

The management model that combines “bottom-up” and “top-down” approaches is emphasized in community disaster risk management. The concept of risk governance focuses on the construction of adaptive governance systems through governance institutions, mechanisms, and methods. By integrating the concepts of community disaster risk management and risk governance with the political context and actual practices of community epidemic prevention and control in China, this study proposes to establish a Chinese community resilience governance system framework that combines “bottom-up” and “top-down” approaches with active participation from residents.

The rapid identification, quick and accurate prediction, and timely alerting of risks are prerequisites for community resilience governance (53). Quantitative and qualitative results in this result show that improved risk perception has a great significance on community resilience. In community resilience governance framework, it is necessary to construct a network-based risk governance bodies that involves various stakeholders such as governments, businesses, social organizations, grassroots communities, and residents (54). The mutual assistance among community residences and substantial input of community epidemic prevention resources during the prevention and control of this epidemic fully highlight the importance of multi-subject participation in a network structure. Community resilience relies on the solid support provided by sound mechanisms and systems. Emergence plans of community disaster management constitute vital support for community resilience (55), while community disaster emergency culture exerts a subtle influence on the behaviors of organizations and members (56). In addition, community resilience governance reflects the flexible adjustments of communities in response to complex changes in external environments, including preparedness, response, and recovery (57). In the prevention and control of this epidemic, prevention, and control measures adopted by the Chinese government during different stages of epidemic occurrence and development, the unity of all Chinese people in fighting the epidemic, and the rapid recovery of community production and livelihood are all the best manifestations of community resilience emergency mechanisms and systems.

### Limitation

Besides the contributions discussed above, this study did have at least three limitations. First, limited by geographical location during this specific period in 2022, this study has employed a simple sampling method. This survey method may have excluded aged people with relatively low technological literacy. Therefore, this study has planned to carry out a multi-stage survey evaluation, in which online and offline surveys will be comprehensively employed, with a method combining random sampling with convenient sampling. Second, in terms of disaster risk reduction behaviors, previous studies mostly focused on such factors as disaster experiences, emergency supply stockpiling, community voluntary service participation, emergency drill practicing, and relevant disaster training and education participation. Based on actual situations, this study has selected three items of disaster risk reduction related to the current pandemic, which could result in a problem of insufficient factors considered. Future studies should further investigate relevant behaviors of disaster prevention and mitigation. Finally, constrained by objective factors including capital, manpower, and material availability, and considering the diversity of geographical locations and levels of social-economic development, three representative provinces have been chosen for inclusion in this study. Nevertheless, potential issues related to underrepresentation may exist. Subsequent research endeavors should incorporate a wider array of regions to enhance the validity of our conclusions.

### Conclusion

This study has conducted a dynamic assessment of community resilience levels in China and analyzed the influencing factors of current community resilience. The results show that after the outbreak of the COVID-19 pandemic, community resilience in China has undergone significant changes, with the community resilience level during the pandemic significantly higher than that in 2015. However, community capital was, is, and will be a weak link and key focus for the building of community resilience. In addition, disaster risk awareness and disaster risk reduction behaviors of residents are two primary factors influencing community resilience in the context of the pandemic. Therefore, it is recommended that future studies should further perform multi-stage evaluations on the changing trend of community resilience and identify specific focuses of community resilience building under different stages. Furthermore, the causal relationship of risk awareness and disaster prevention and mitigation activities with enhanced community resilience should be further explored.

## Data availability statement

The raw data supporting the conclusions of this article will be made available by the authors, without undue reservation.

## Ethics statement

This research was approved by the Institutional Review Board of Harbin Medical University. All participants were informed about the purpose of this study in advance, and data were collected voluntarily.

## Author contributions

CY: Data curation, Methodology, Writing – original draft. XL: Data curation, Writing – review & editing. NZ: Data curation, Writing – review & editing. YL: Data curation, Writing – review & editing. BW: Methodology, Writing – review & editing. CS: Methodology, Writing – review & editing. YT: Methodology, Writing – review & editing. YQ: Data curation, Writing – review & editing. BY: Data curation, Writing – review & editing. LZ: Methodology, Writing – review & editing. NN: Conceptualization, Funding acquisition, Resources, Supervision, Validation, Writing – review & editing.

## Footnotes

^1^www.wjx.cn
